# Polypharmacy in Polish Older Adult Population—A Cross-Sectional Study: Results of the PolSenior Project

**DOI:** 10.3390/ijerph19031030

**Published:** 2022-01-18

**Authors:** Agnieszka Neumann-Podczaska, Slawomir Tobis, Demetra Antimisiaris, Malgorzata Mossakowska, Monika Puzianowska-Kuznicka, Jerzy Chudek, Lukasz Wierucki, Piotr Merks, Barbara Wizner, Malgorzata Sobieszczanska, Zofia I. Niemir, Beata Kaczmarek, Katarzyna Wieczorowska-Tobis

**Affiliations:** 1 Geriatric Unit, Department of Palliative Medicine, Poznan University of Medical Sciences, 61-245 Poznan, Poland; ar-n@wp.pl (A.N.-P.); be.kaczmarek@gmail.com (B.K.); kwt@tobis.pl (K.W.-T.); 2 Department of Occupational Therapy, Poznan University of Medical Sciences, 60-781 Poznan, Poland; 3 Frazier Polypharmacy Program, University of Louisville, Louisville, KY 40292, USA; deanti01@louisville.edu; 4 International Institute of Molecular and Cell Biology, 02-109 Warsaw, Poland; malgosia@iimcb.gov.pl; 5 Department of Human Epigenetics, Mossakowski Medical Research Institute, Polish Academy of Sciences, 02-106 Warsaw, Poland; mpuzianowska@imdik.pan.pl; 6 Department of Geriatrics and Gerontology, Medical Centre of Postgraduate Education, 01-813 Warsaw, Poland; 7 Department of Internal Medicine and Oncological Chemotherapy, Faculty of Medical Sciences in Katowice, Medical University of Silesia, 40-027 Katowice, Poland; chj@poczta.fm; 8 Department of Preventive Medicine, Medical University of Gdansk, 80-210 Gdansk, Poland; wierucki@gumed.edu.pl; 9 Collegium Medicum, Faculty of Medicine, Cardinal Stefan Wyszyński University, 01-938 Warsaw, Poland; piotrmerks@googlemail.com; 10 Department of Internal Medicine and Gerontology, Jagiellonian University Medical College, 30-688 Krakow, Poland; barbara.wizner@uj.edu.pl; 11 Department and Clinic of Geriatrics, Wroclaw Medical University, 50-369 Wroclaw, Poland; malgorzata.sobieszczanska@umed.wroc.pl; 12 Department of Nephrology, Transplantology and Internal Diseases, Poznan University of Medical Sciences, 60-355 Poznan, Poland; zniemir@ump.edu.pl

**Keywords:** PolSenior, polypharmacy, excessive polypharmacy, older adults, correlates

## Abstract

Polypharmacy is a challenging issue in geriatrics. The aim of the study was to characterize correlates of polypharmacy in the PolSenior project. The PolSenior project, was a comprehensive survey in a large and longitudinal representative sample of thePolish older population. The project was conducted by the International Institute of Molecular and Cell Biology in Warsaw between 2008 and 2011. All medications consumed during the week preceding the survey were evaluated for each participant (n = 4793, including 2314 females (48.3%)). Thereafter, the percentage of those with polypharmacy (at least 5 medications) and excessive polypharmacy (at least 10 medications) was calculated, and their correlates were determined. The average number of medications used by participants was 5.1 ± 3.6, and was higher in females than in males (5.5 ± 3.5 vs. 4.8 ± 3.5; *p* < 0.001). Polypharmacy characterized 2650 participants (55.3%) and excessive polypharmacy—532 of them (11.1%). The independent correlates associated withpolypharmacy were: age over 70 years, female sex, higher than primary education, living in an urban area, comorbidities, any hospitalization during past five years, and visiting general practicioners at least yearly. As for correlates with excessive polypharmacy, they were: age 80–84 years, female sex, living in an urban area, diagnosis of at least four chronic diseases, and at least two hospitalizations in the last five years. This study serves as a starting place to understand patient characteristics associated with polypharmacy, excessive polypharmacy, and identify targeted interventions.

## 1. Introduction

Multimorbidity is highly prevalent in older adults [1] and is typically accompanied by multiple drug regimens, described as polypharmacy. As there are various definitions of polypharmacy (reviewed by Fulton et al. [2]), it is challenging to compare its prevalence. In a nationally representative sample of Korean older patients [3], polypharmacy was defined as the concurrent use of six or more medications, and was present in as many as 86.4% of studied subjects. Notably, the prevalence was almost half as common (44%) in a registry-based prospective cohort study from Sweden, even though polypharmacy was defined as concurrent use of five or more medications (versus six in the Korean study) [4]. Despite these differences, the prevalence of polypharmacy generally increases over time. Jyrkka et al. [5] reported that in Finland, between 1998 and 2003, in a cohort of older adults, polypharmacy prevalence (>5 medicines in use) increased from 54% to 67%, and excessive polypharmacy (≥10 medications in use) increased from 19% to 28%. In more recent times, Swedish nationwide registry-based study by Zhang et al. [6] reported that the prevalence of polypharmacy (≥5 medications) and excessive polypharmacy (≥10 medicines in use) significantly increased between 2006 and 2014. The authors of this study stated that the prevalence of polypharmacy and excessive polypharmacy increased radically with age and peaked up to 79.6% and 36.4% in individuals aged 90 and above, respectively. These upward trends in polypharmacy may have many unpredictable effects [7]. One trend for certain is that polypharmacy is related to adverse clinical outcomes [8]. It was also shown that medication adherence is negatively associated with greater number of mediactions [9]. Several previous studies have reported that polypharmacy was associated with increased incidence of adverse drug reactions, drug–drug interactions, and inappropriateness of pharmacological treatment [10,11,12]. Moreover, polypharmacy is linked to poorer health status as it increases the risk of geriatric syndromes such as falls [13], malnutrition [14], urinary incontinence [15], and depression [16]. Additionally, it has been established that inappropriately prescribed polypharmacy leads to functional impairment and increases mortality risk [17,18]. Consequently, polypharmacy contributes to an increase in both direct and indirect costs of medical care [19]. Furthermore, polypharmacy regimens constitute a well-known risk factor of non-adherence [9]. According to Pasina et al. [20], an increased number of medications at hospital discharge strongly correlates to non-adherence, which averages to as much as 69.6% among community-dwelling older subjects three months after discharge.

To address the challenges resulting from polypharmacy and its adverse outcomes, frameworks for various interventions are proposed. According to the Canadian Agency for Drugs and Technologies in Health (CADTH), several types of interventions directed at different health system stakeholders can reduce the polypharmacy burden among older individuals [21]. They include those targeting health professionals, drug consumers, as well as organizational, financial, and regulatory interventions, which aim to change the delivery of health services. All of these interventions require continuous actions aimed at identifying and solving medication-related problems to decrease the risk of polypharmacy and increase the efficacy and safety of treatment [22].

Fifteen years ago, Zarowitz et al. demonstrated the positive effect of a teamwork intervention, including a physician, a pharmacist, and the patient, on the reduction in polypharmacy in community-dwelling older subjects [23]. Currently, there is also a large body of evidence on the effectiveness of various interventions in reducing the risks associated with polypharmacy. The studies by Cooper et al. [24] and Olaniyan et al. [25] showed that both professional, organizational, and multifaceted interventions resulted in the reduction in potentially inappropriate prescriptions and the improvement of adherence, appropriateness, and safety of medications.

Given that polypharmacy is associated with adverse health outcomes and can be reduced by applying different methods of medication revision, it is vital to define the subjects who are likely to benefit most from such interventions and find the predictors of polypharmacy. Thus, the aim of the study was to investigate the prevalence of polypharmacy in older individuals in Poland and to delineate the predictors of polypharmacy within a nationwide representative study group. A similar comprehensive population-based study has not been published to date to the best of our knowledge.

## 2. Materials and Methods

PolSenior was a nationwide multidisciplinary project conducted by International Institute of Molecular and Cell Biology in Warsaw between 2008 and 2011 on a group representative of the Polish population of older adults to assess medical, psychological, social, and economic aspects of aging in Poland. Therefore, the aim of the project was to define the status of study participants and identify their social and medical needs. The outcomes were expected to facilitate the establishment of proper care for the growing population of older individuals [26].

There were 4979 older people included in the project (2412 women and 2567 men) aged 65 years and older. A detailed description of the study design has been published and can be found at https://doi.org/10.1016/j.exger.2011.09.006 (accessed on 1 December 2021) [26].

Analysis of pharmacotherapy was performed among 4793 participants (including 2314 women—48.3% of the total, and 2479 men). In 186 participants (3.7% of the total study group), analysis of pharmacotherapy was not possible due to a lack of information on participants’ medication regimens.

For each of the studied participants, the evaluation of all pharmaceutical preparations (including dietary supplements, complementary/alternative medicines, and herbal remedies), referred to as “medications,” consumed during the week preceding the survey was performed. The interviewer wrote down the name, the formulation (e.g., tablets, capsules, drops), the single dose, and the frequency of dosage of each medication that was taken more than once a week. Information on pharmacotherapy was obtained directly from the respondent or their family/caregivers, who were also asked to provide a “brown bag” containing all consumed pharmaceutical preparations to the interviewer.

Each medicine taken by the respondent was coded according to the alphanumeric Anatomical Therapeutic Chemical (ATC) classification system. Quantitative analysis of the medication involved the calculation of the average number of all medications (both prescribed, Rx and over-the-counter, OTC) consumed by the studied subjects. The percentages of respondents (by gender) not taking any medication and of those receiving five or more and ten or more medications in the last week were calculated.

The only morbidities (disease states) included in the analysis were cardiovascular diseases (including hypertension, coronary heart disease, myocardial infarction, and stroke), respiratory, digestive, endocrine, and metabolic diseases (including diabetes mellitus), blood diseases (anemia), kidney diseases, osteoporosis, and eye diseases.

To delineate the non-biomedical health predictors of polypharmacy, the following variables were taken into account: age, sex, marital status (unmarried/married or in a relationship), education (less than primary, primary, vocational, at least secondary), place of residence (city/rural area), living conditions (alone/with others/in an institution), number of diseases (0/1–3, or at least 4), number of hospitalizations during the last 5 years (0/1 or 2 or more than 2), frequency of general practicioners consultations (less than once a year/several times a year/at least once a month), and self-reported poverty (YES—if they could not afford to buy even the most inexpensive food and clothing).

### Statistical Analysis

The statistical analysis was performed with STATISTICA 13.0 software by TIBCO Software (Palo Alto, CA, USA). For the analyzed variables, mean values and standard deviations were calculated. Normality in the distribution of variables was assessed with the Shapiro–Wilk test. Comparison between two unpaired groups was made with the Mann–Whitney test and the Kruskal–Wallis test for more than two groups. In the case of significant differences between studied variables detected by the Kruskal–Wallis test, a post hoc Dunn test was performed. Statistical significance of differences in the distribution of quality variables between two or more groups was analyzed with the χ^2^ test.

To assess simultaneous interdependence between many variables, a multiple regression model (logistic regression) was used, specifying the odds ratio and the confidence interval with the confidence limit of 95%. All variables that were significant for polypharmacy and excessive polypharmacy in the univariable analysis were included in multiple linear regression analysis. *p* < 0.05 was considered statistically significant.

The Polsenior project sample was intended to include balance in the total numbers of participants as well as balanced numbers of men and women in all age cohorts, which allowed for precise assessment of studied factors in the oldest groups. However, it caused an overestimation of older groups and men in terms of the population structure. Consequently, in order to make the sample representative of the Polish population and obtain results reflecting the distribution of studied characteristics in the entire population of older people in Poland, post-stratification was necessary.

## 3. Results

Of the 4793 respondents with a mean age of 79.3 ± 8.7 years, 2314 were women (48.3%). There was no difference in age between gender groups, of females and males (79.2 ± 8.9 and 79.4 ± 8.5, respectively). However, studied females had lower education than males (*p* < 0.0001), more often lived alone (*p* < 0.0001) and reported poverty less common than males (*p* < 0.0001). Table 1 shows detailed characteristics of the studied group, including gender.

The mean number of comorbidities was 2.1 ± 1.6, and the mean number of hospitalizations—1.3 ± 2.0. The number of comorbidities was higher in females (2.3 ± 1.7 vs. 2.0 ± 1.5; *p* < 0.001), whereas the number of hospitalization was higher in in males (1.3 ± 2.0 vs. 1.2 ± 2.0; *p* < 0.01). The mean number of medications used by studied respondents was 5.1 ± 3.6. Females took more medications than males (5.5 ± 3.5 vs. 4.8 ± 3.5; *p* < 0.001).

**Figure 1:**
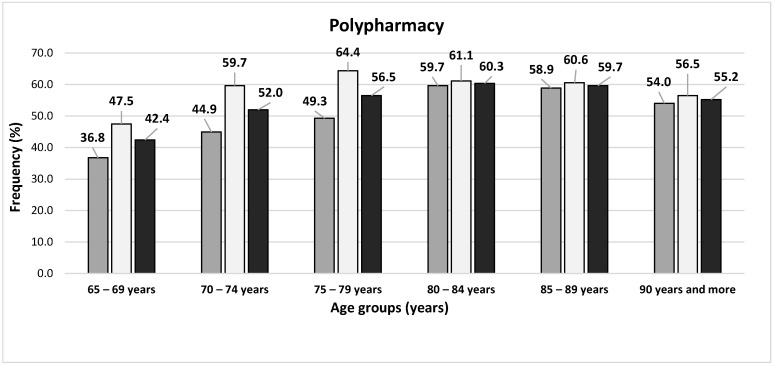

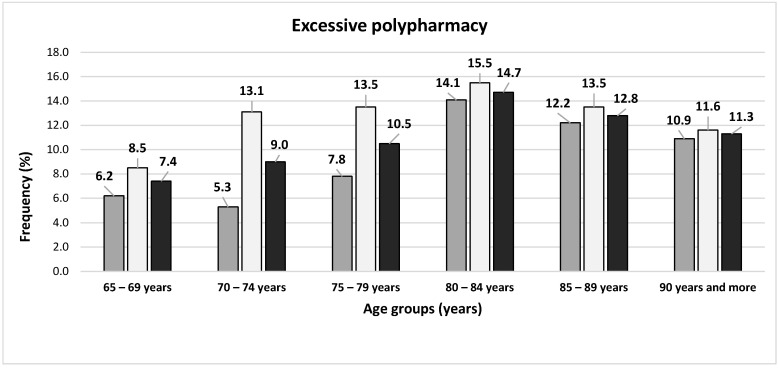
The prevalence of polypharmacy and excessive polypharmacy in this study sample of the Polish older adult population, including gender: dark grey bars represent males, light grey bars—females, and black bars—the whole studied population.

Among studied subjects, 507 (10.4%) did not take any medication, and more common inmales than females (331; 13.1% vs. 176; 7.4%, *p* < 0.0001). At least five medications were taken regularly by 2650 subjects (54.4%) and at least 10—by 532 (10.9%). Both polypharmacy and excessive polypharmacy were more common in females than males (58.2% vs. 50.8%, *p* < 0.0001 and 12.6% vs. 9.4%, *p* = 0.0003, respectively). Figure 1 presents the frequency of polypharmacy and excessive polypharmacy in age cohorts, including gender.

### The Participant Characteristics with Excessive Polypharmacy

In univariable analysis, the impact of single variables on the incidence of excessive polypharmacy among those with polypharmacy is presented in Table 2. The rate of excessive polypharmacy was the highest among subjects aged 80–84 (24.4%) and the lowest in the two youngest cohorts (60–64 years and 65–69 years: 17.4%). Subjects living in urban areas had excessive polypharmacy more often than those living in rural areas (*p* < 0.0001).

As far as biomedical health-related characteristics associated with polypharmacy are concerned, subjects with at least four chronic diseases consumed at least ten medications more frequently, compared with those with no chronic diseases (*p* < 0.001, Table 3). Moreover, excessive polypharmacy was more common in those who were hospitalized at least twice in the last five years in comparison with the subjects not hospitalized at all (*p* < 0.001).

The multiple regression model established the following associations with excessive polypharmacy: age 80–84 years, female sex, living in an urban area, diagnosis of at least four chronic diseases, and at least two hospitalizations in the last five years. The detailed results are presented in Table 4.

## 4. Discussion

The growing incidence of polypharmacy among older adults and the associated adverse health consequences make the search for its correlates of particular importance to characterize those at risk of its occurrence. To the best of our knowledge, this is the first observational, nationwide study describing comprehensive associations with polypharmacy and exploring the potential determinants of polypharmacy with unique data collection. We asked older individuals or their caregivers to show us all pharmaceutical preparations being consumed, which allowed for real insight into the treatment used, including OTC medications and dietary supplements/natural health products/alternative medicines. Such preparations often remained unmentioned by older people in various surveys and are sometimes not captured in electronic medical records and claims data. However, their potential interactions and adverse drug reactions can be significant, especially within a multi-medication regimen [27].

Our study indicated that over 50% of older individuals took at least five medications, and more than 10% took at least ten. In the SHARE project, based on a representative sample of community-based older populations from 17 European countries, the prevalence of concurrent use of at least five medications ranged from 26.3% to 39.9% (in Poland, 33.8%) [28]. However, the methodology of data collection used in the SHARE project (i.e., simple question whether the number of medications taken is at least 5) justifies the differences observed in relation to our study, because the SHARE participants may not have included all of the pharmaceutical preparations consumed (supplements, etc.). In older subjects, the main reason for not providing information on taking alternative medicines is failing to ask a directional question about it [29]. The SHARE study also did not include excessive polypharmacy, greater than ten products.

Analyses regarding polypharmacy are often based on national registries, so they only include prescription medications, and in addition, as each country has its own rules for medications reimbursement lists (claims data), the possibilities for comparing results are limited. According to a data registry-based study from Sweden, the prevalence of polypharmacy was higher than in our study, at 44.0%, and excessive polypharmacy at 11.7% [4].

From a methodological point of view, the study of Walckiers et al. [30] is similar to ours, based on the Belgian Health Interview Survey, in which respondents showed medicines to the interviewer. However, the comparison is also difficult in this case because the analysis considered only medications listed in the Belgian pharmacopeia. Notably, despite adopting a lower cut-off point for excessive polypharmacy in the Belgian study (at least nine medications), its frequency was lower than in our assessment (8.2%).

The fact that we included all the preparations consumed appears to be relevant to some of the relationships we observed. It is known that among individuals with higher education, there is a greater tendency to self-treatment [31]. They thus often add OTC medications and dietary supplements, natural health products, and alternative medicines to their medication regimen, which increases the polypharmacy level. It seems possible that this phenomenon had its significance in our study for the observed association of polypharmacy with a higher-than-primary level of education. However, one may speculate that the preparations added to pharmacotherapy in self-treatment are not so numerous as to cause an increase in the number of medications taken to ten and more. Moreover, we did not observe an association of self-treatment and the use of ten or more medications with education.

As for people with higher income, it can be expected that they more often use OTC preparations (e.g., medications advertised as aging modifiers or dietary supplements). In turn, an earlier analysis of general practicioners’ data suggests that more money is spent on prescriptions for those with a lower socioeconomic position because it is known that social determinants of health (to which low income and poverty belong) impact higher disease burdens, which may, in turn, cause higher polypharmacy [32]. The overlapping of these two phenomena may lead to the observed lack of relationship between polypharmacy and income.

In our study—as in many others—the female sex was associated with both polypharmacy and excessive polypharmacy. Women’s life expectancy is longer than men’s, which predisposes them to chronic comorbidities that are present for a longer period of their lives, and could have some impacted our results. Women’s attention to their health status (more pronounced than with men) results in a higher frequency of health consultations [33]. Women appear to be more likely to report both symptoms and complaints [34] and take part in preventive health care initiatives. This makes them more likely to be prescribed and self-treated with a higher number of primary and secondary prevention medicines.

As far as age (as a risk factor of polypharmacy) is concerned, we found the so-called survivor effect, as the oldest old had no increased severity of polypharmacy (nor excessive polypharmacy) observed. This is in agreement with previous studies, which suggested an inverted association between age and the number of medications after the age of 85 years [35]. Our findings are also consistent with other branches of the PolSenior projects, pointing to decreased prevalence of hypertension and pain in the oldest old. The less frequent occurrence of these conditions in the most senior group also suggests lower use of cardiovascular and analgesic medications, which are the categories of medications most commonly taken by older adults [36].

Among the socio-demographic correlates of polypharmacy, living in an urban area predisposed to both polypharmacy and excessive polypharmacy. Similar results were found in Chinese residents by Yang et al. [37]. They suggested that higher drug consumption among urban residents was due to the fact that they are more likely to have numerous doctors’ appointments and more often use self-treatment. Likewise, in Poland, access to health care (both primary and specialist) is easier in urban than in rural areas. This applies equally to the access to pharmacies, and thus also to pharmaceutical advice and preparations available in self-treatment, which can considerably contribute to the increase in polypharmacy among the city residents we describe.

We found an association of polypharmacy and excessive polypharmacy with a greater number of diseases. Multimorbidity is a common feature in geriatrics about which there is an agreement that it translates into an increase in the number of medications used [38,39]. Not surprisingly, polypharmacy was also associated with a higher number of hospitalizations within the past five years. Walckier et al. also showed an association between multiple drug prescriptions and inpatient hospitalizations in the past 12 months [30].

Notably, in our study, polypharmacy (but not excessive polypharmacy) was related to a high number of general practicioners’ consultations. One can speculate that patients with substantial multimorbidity are frequently consulted by specialists, which increases thepolypharmacy level. Our observations complement the study of Walckiers et al. [30], who claimed that visits to specialists in the past two months were associated with excessive polypharmacy only and not so with (lower level) polypharmacy. A possible explanation for this phenomenon is that patients with polypharmacy are consulted more often by general practicioners, whereas those with excessive polypharmacy are additionally consulted by specialists.

In our study, excessive polypharmacy was also related to living in an institution. According to a study conducted in nursing homes in eight European countries, 24% of residents were exposed to excessive polypharmacy [39]. Moreover, a prospective cohort study conducted in Sweden proved that living in a nursing home not only was associated with an increased risk of excessive polypharmacy at baseline but also with the incidence of excessive polypharmacy in the future [4]. It is therefore likely that multimorbidity, as well as lower functional status of nursing home residents, causes the number of preparations to quickly reach or even exceed the landmark number of ten for excessive polypharmacy.

Our study has some limitations. The study was cross-sectional thus it can not imply causality. The participation rate in the PolSenior project was not very high (49%), which may have affected the findings. To assess the association between polypharmacy and the number of diseases, only selected conditions were taken into account; however, such an approach was also applied by other authors [40]. The strength of the study was the methodology of collecting data and the high number of the oldest participants, including men. All questionnaires were administered by trained staff. Data on drug consumption were not obtained via a simple questionnaire in which the respondent is only verbally asked about medications taken. This method is potentially inaccurate since the participant may not remember all medications taken. As far as we know, this is the only study where the methodology of ‘brown bag session’ was applied throughout, and all pharmaceutical preparations (not only prescribed medicines) were presented by the participant or caregiver to the interviewer.

In summary, polypharmacy associated with hospitalization and institutionalization has the highest possibility of modification through efforts to improve medication management and deprescribing in hospital and institutional settings. Therefore, in order to reduce the risk of polypharmacy and related (potential) adverse outcomes, focusing on the impact of hospitalization and institutionalization on polypharmacyshould yield measurable results. In a Swiss study of 900 older inpatients of an internal medicine ward, the introduction of an easy-to-use checklist aimed at supporting the therapeutic decision of physicians significantly reduced the risk of prescribing unnecessary medications at discharge [41]. In regard to nursing home settings, it was also shown that an effective geriatrician-led intervention based on consultations offered to patients’ primary physicians substantially reduced polypharmacy [42].

## 5. Conclusions

The results of our study indicate that the prevalence of polypharmacy is high in the Polish older population. To assess the actual incidence and define the determinants of the occurrence of analyzed phenomena, comprehensive methodology of data collection is essential, including all pharmaceutical preparations consumed (both prescribed and OTC), with alternative medicines, dietary supplements, herbal remedies, etc.

With the aging population and increased availability of medicines, polypharmacy will become an escalating health problem in the coming years. To assure the effectiveness and safeness of pharmacotherapy, it is essential to find a balance between adequate control of diseases and avoiding unnecessary multi-medication regimens. To reduce the risk of polypharmacy, multifaceted interventions based on medication use review and deprescribing strategies as well as organizational interventions, with targeted prescribers both within the hospital and nursing home settings, are needed. This study serves as a starting place to understand patient characteristics associated with polypharmacy and excessive polypharmacy for targeted interventions.

## Figures and Tables

**Table 1:** The characteristics of study participants including sex (base rate: % of line).

				Sex				
Variable				Males		Females		
		n	%	n	%	n	%	*p*
Age (years)	65–69	772	15.8%	372	48.2%	400	51.8%	
	70–74	908	18.6%	474	52.2%	434	47.8%	
	75–79	827	17.0%	434	52.5%	393	47.5%	
	80–84	774	15.9%	412	53.2%	362	46.8%	0.1040
	85–89	846	17.4%	460	54.4%	386	45.6%	
	at least 90	746	15.3%	367	49.2%	379	50.8%	
Marital status	Unmarried	2324	49.4%	737	31.0%	1643	69.0%	<0.0001
	Married	2380	50.6%	1699	73.1%	625	26.9%	
Education	Less than primary	653	13.9%	262	40.1%	391	59.9%	
	Primary	2080	44.2%	955	45.9%	1125	54.1%	<0.0001
	Vocational	906	19.2%	488	53.9%	418	46.1%	
	At least secondary	1069	22.7%	735	68.8%	334	31.2%	
Place of residence	Urban area	2933	60.2%	1547	52.7%	1386	47.3%	0.0756
	Rural area	1940	39.8%	972	50.1%	968	49.9%	
Living conditions	Alone	1008	21.4%	354	35.1%	654	64.9%	
	With others	3660	77.6%	2063	56.4%	1597	43.6%	<0.0001
	In institutions	46	1.0%	24	52.2%	22	47.8%	
Number of diseases	0	416	10.6%	272	65.4%	144	34.6%	
	1–3	2853	73.0%	1496	52.4%	1357	47.6%	<0.0001
	4 or more	639	16.4%	291	45.5%	348	54.5%	
Number of hospitalizations during past 5 years	0	2087	45.7%	1028	49.3%	1059	50.7%	
	1	1111	24.4%	596	53.7%	515	46.4%	0.0033
	2 or more	1366	29.9%	747	54.7%	619	45.3%	
Frequency of GP consultation	Less than once a year	736	16.3%	429	58.3%	307	41.7%	
	Several times a year	1470	32.4%	735	50.0%	735	50.0%	0.0003
	At least ones a month	2327	51.3%	1171	50.3%	1156	49.7%	
Self-reported poverty	NO	3747	88.4%	2026	54.1%	1721	45.9%	<0.0001
	YES	490	11.6%	201	41.0%	289	59.0%	

Note: The numbers in the table may not add up to the total number of participants in the survey, as not all participants answered all the survey questions.

**Table 2:** Study participant characteristics and prevalence of polypharmacy and excessive polypharmacy, in this sample of the Polish older adult population.

Variable		Number of Medications					Number of Medications				
		1–4		5+		*p*	5–9		10+		*p*
Age (years)	65–69	330:	50.2%	327:	49.8%	<0.0001	270:	82.6%	57:	17.4%	0.0693
	70–74	331:	41.2%	472:	58.8%		390:	82.6%	82:	17.4%	
	75–79	287:	38.1%	467:	61.9%		380:	81.4%	87:	18.6%	
	80–84	244:	34.3%	467:	65.7%		353:	75.6%	114:	24.4%	
	85–89	269:	34.8%	505:	65.2%		397:	78.6%	108:	21.4%	
	At least 90	255:	38.2%	412:	61.8%		328:	79.6%	84:	20.4%	
Marital status	Married	861:	42.0%	1190:	58.0%	0.0010	1075:	80.8%	289:	19.2%	0.2213
	Unmarried	801:	37.0%	1364:	63.0%		962:	78.8%	228:	21.2%	
Education	Less than primary	228:	40.7%	332:	59.3%	0.0003	257:	82.8%	57:	17.2%	0.0574
	Primary	781:	42.1%	1073:	57.9%		866:	80.7%	207:	19.3%	
	Vocational	281:	33.4%	561:	66.6%		424:	75.6%	137:	24.4%	
	At least secondary	374:	38.8%	591:	61.2%		474:	80.2%	117:	19.8%	
Place of residence	Urban area	964:	35.8%	1728:	64.2%	<0.0001	1336:	77.3%	392:	22.7%	<0.0001
	Rural area	752:	44.9%	922:	55.1%		782:	84.8%	140:	15.2%	
Living conditions	Alone	321:	34.8%	602:	65.2%	0.0050	479:	79.6%	123:	20.4%	0.1805
	With others	1326:	40.7%	1932:	59.3%		1546:	80.0%	386:	20.0%	
	In istitutions	18:	40.9%	26:	59.1%		17:	65.4%	9:	34.6%	
Number of diseases	0	197:	69.1%	88:	30.9%	<0.0001	79:	89.8%	9:	10.2%	<0.0001
	1–3	1124:	43.6%	1455:	56.4%		1203:	82.7%	252:	17.3%	
	4+ or more	111:	17.7%	517:	82.3%		367:	71.0%	150:	29.0%	
Number of hospitalizations during past 5 years	0	912:	52.5%	826:	47.5%	<0.0001	693:	83.9%	133:	16.1%	<0.0001
	1	403:	39.2%	625:	60.8%		517:	82.7%	108:	17.3%	
	2 or more	300:	22.7%	1022:	77.3%		761:	74.5%	261:	25.5%	
Frequency of general practioners’ consultation	Less than once a year	305:	62.4%	184:	37.6%	<0.0001	156:	84.8%	28:	15.2%	*p* = 0.1672
	Several times a year	591:	43.4%	770:	56.6%		617:	80.1%	153:	19.9%	
	At least once a month	697:	31.0%	1553:	69.0%		1226:	78.9%	327:	21.1%	
Self-reported poverty	NO	1327:	39.4%	2037:	60.6%	0.6314	1626:	79.8%	411:	20.2%	*p* = 0.6960
	YES	167:	38.1%	271:	61.9%		213:	78.6%	58:	21.4%	

Note: The numbers in the table may not add up to the total number of participants in the survey, as not all participants answered all the survey questions.

**Table 3:** Multi regression analysis of polypharmacy correlates in this sample of the Polish older adult population.

		Number of Medications: 1–4 vs. 5+		
Variable		OR	95% CI	*p*
Sex	Males	-	-	-
	Females	1.35	1.14–1.62	0.001
Age (years)	65–69	-	-	-
	70–74	1.48	1.15–1.91	0.002
	75–79	1.58	1.22–2.06	0.001
	80–84	2.01	1.52–2.66	<0.001
	85–89	2.34	1.76–3.10	<0.001
	At least 90	2.12	1.62–2.98	<0.001
Marital status	Married	-	-	-
	Unmarried	0.91	0.74–1.12	0.37
Education	Less than primary	0.96	0.74–1.23	0.729
	Primary	-	-	-
	Vocational	1.43	1.15–1.79	0.001
	At least secondary	1.32	1.07–1.63	0.010
Place of residence	City	1.30	1.09–1.54	0.003
	Rural area	-	-	-
Living conditions	Alone	1.20	0.95–1.50	0.121
	With others	-	-	-
	In istitutions	1.11	0.45–2.71	0.826
Number of diseases	0	-	-	-
	1–3	2.41	1.80–3.22	<0.001
	4 or more	6.75	4.70–9.69	<0.001
Number of hospitalization during past 5 years	0	-	-	-
	1	1.49	1.23–1.79	<0.001
	2 or more	2.52	2.08–3.06	<0.001
Frequency of general practiocioners’ consultation	Less than once a year	-	-	-
	Less than once a year	1.70	1.32–2.20	<0.001
	At least once a month	2.88	2.25–3.68	<0.001

**Table 4:** Multi regression analysis of excessive polypharmacy correlates in the PolSenior project.

Variable		Number of Medications: 5–9 vs. 10+		
		OR	95% CI	*p*
Sex	Males	-	-	-
	Females	1.39	1.09–1.77	0.007
Age (years)	65–69	-	-	-
	70–74	0.95	0.62–1.44	0.794
	75–79	0.99	0.64–1.51	0.953
	80–84	1.53	1.01–2.33	0.044
	85–89	1.50	0.98–2.28	0.062
	At least 90	1.32	0.84–2.09	0.224
Education	Less than primary	0.74	0.49–1.12	0.149
	Primary	-	-	-
	Vocational	1.32	0.98–1.79	0.070
	At least secondary	1.09	0.80–1.49	0.599
Place of residence	City	1.35	1.03–1.77	0.029
	Rural area	-	-	-
Number of diseases	0	-	-	-
	1–3	1.64	0.80–3.35	0.175
	4 or more	2.91	1.39–6.07	0.004
Number of hospitalizations during past 5 years	0	-	-	-
	1	0.96	0.70–1.33	0.818
	2 or more	1.55	1.17–2.04	0.002
